# Association of PFDeA exposure with hypertension (NHANES, 2013–2018)

**DOI:** 10.1038/s41598-024-51187-4

**Published:** 2024-01-09

**Authors:** Jie Li, Suling Ye, Zeyuan Zhao, Zhao Xue, Shupeng Ren, Yue Guan, Chuang Sun, Qiying Yao, Liang Chen

**Affiliations:** 1 Department of Cardiology, The Second Affiliated Hospital of Dalian Medical University, Dalian, China; 2 Department of Physiology, Dalian Medical University, Dalian, China

**Keywords:** Environmental sciences, Cardiology, Health care, Medical research

## Abstract

Perfluoroalkyl and polyfluoroalkyl substances (PFASs) is a series of artificial compounds which is associated with human health. However, there are few studies on the relationship between PFASs and hypertension. In this study, we examined the association between different kinds of PFASs and hypertension. Multivariable logistic regression and subgroup analysis were adopted to assess the associations between PFASs and hypertension. Spline smoothing plots and linear regression were used to assess the relationship between PFASs and blood pressure. We found a positive association between serum PFDeA concentrations and the prevalence of hypertension after fully adjusting confounders (OR = 1.2, P = 0.01), but other types of PFASs showed no positive results. Subgroup analysis stratified by ethnicity showed there was a stronger relationship among non-Hispanics than Hispanics. Serum PFDeA concentrations were positively associated with systolic pressure (β = 0.7, P< 0.01) and diastolic blood pressure (β = 0.8, P< 0.01) among non-Hispanics who did not take antihypertensive drugs. This study showed that PFDeA exposure was associated with hypertension in Americans who identify as non-Hispanic. There was a positive association between PFDeA and blood pressure in non-Hispanic Americans who did not take antihypertensive drugs.

## Introduction

Perfluoroalkyl and polyfluoroalkyl substances (PFASs) is a general term for a series of artificial compounds widely used in consumer goods and industrial products, including disposable food packaging, cookware, surfactants, lubricants, carpets, and fire-retarding foam^1^. The forms of these PFASs are so stable that it is difficult for them to degrade naturally in the environment, which has led to their accumulation in the environment since the onset of production in the late 1940s^2^. Because these compounds exist widely, humans are exposed to PFASs in many aspects of their lives, including in drinking water, seafood, indoor air, and other pollutants^1,3–5^. In recent years, exposure to PFASs has been proven to affect human health^6–10^.

Hypertension is one of the most important risk factors for cardiovascular diseases^11^, the occurrence of which is related to various factors, including genes, diet, obesity, living habits, and so on^12,13^. In recent years, increasingly more studies have reported that environmental pollutants can also lead to hypertension^14–16^.

Some studies have reported that PFAS exposure is related to the occurrence of pregnancy-induced hypertension^17–19^. One study reported that PFAS exposure is related to hypertension, and this association was more significant in women^20^. Another study reported the association between PFASs and hypertension in women^21^. However, a cross-sectional study found that PFASs were only associated with hypertension in men^22^. However, some studies have reported that no association exists between PFASs and hypertension^23–26^. A cross-sectional study found that perfluorooctanoic acid (PFOA), an artificial compound of PFASs, is associated with hypertension^27^. Another study showed that only PFOA and a few compounds of PFASs were related to hypertension, but there was no significant association between other PFASs and hypertension^28^. However, one study reported that PFOA has no association with hypertension^29^. Overall, there is no unified perspective on the relationship between PFAS exposure and hypertension^6^. Moreover, research on the relationship between PFASs and hypertension is limited to several compounds, such as PFOA, and the association between other PFASs and hypertension remains unclear. If the hypothesis that PFASs with longer carbon chains have greater toxicity is accurate, then many types of PFAS are more toxic than PFOA^30^, including perfluorodecanoic acid (PFDeA). However, there has been little research into this hypothesis.

Therefore, we examined the association between different kinds of PFAS exposure and hypertension using the database of the National Health and Nutrition Examination Survey (NHANES) in the United States, including PFDeA, perfluorohexane sulfonic acid (PFHxS), perfluorononanoic acid (PFNA), n-perfluorooctanoic acid (n-PFOA), n-perfluorooctane sulfonic acid (n-PFOS), perfluoromethylheptane sulfonic acid isomers (Sm-PFOS).

## Methods

### Data source and study population

**Figure 1:**
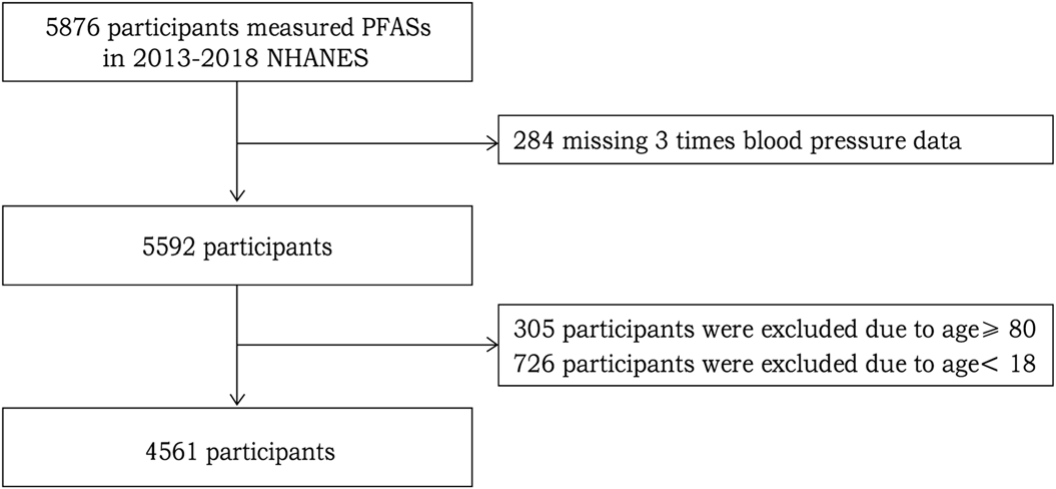
Flowchart of participant selection.

The study data were derived from NHANES, a national survey that monitors the health and nutritional status of Americans. NHANES data can be obtained online (https://www.cdc.gov/nchs/nhanes/index.htm). NHANES enrolled 29,400 participants in from 2013 to 2018. In this study, among participants over 12 years old, one third were selected for the measurement of various types of PFASs. This resulted in a total of 5876 participants in whom we measured concentrations of 6 types of target PFASs. We excluded 1315 participants for the following reasons: 284 lacked blood pressure data, 305 participants were ≥ 80 years old, and 726 were < 18 years old. Finally, 4561 adult participants were included in this study (Fig. 1). The study protocol of NHANES has been approved by the Ethics Review Board of the National Center for Health Statistics Research. All participants provided written informed consent to authorize the use of their data. All experiments involved in this paper are performed in accordance with relevant guidelines and regulations.

### Serum PFAS measurement

One-third of participants aged ≥ 12 years were randomly selected for measurement of serum PFASs in online solid phase extraction coupled to high-performance liquid chromatography-turboionspray ionization tandem mass spectrometry. The detail of laboratory methods can be found in the PFAS Laboratory Procedure Manual of NHANES.

During 2013–2018 in NHANES, the serum concentrations of various PFASs in a group of participants were measured, and nine PFASs were measured in three cycles. Six types of PFASs with valid data (here defined as numerical results higher than the detection limit) that were detected in more than 70% of participants were selected for inclusion in the present study: PFDeA, PFHxS, PFNA, n-PFOA, n-PFOS, Sm-PFOS. All PFASs were recorded as continuous variables. The value was replaced with the detection limit (LOD) divided by the square root of two when it was below the LOD.

### Definition of hypertension

Blood pressure was measured using a standard protocol by trained investigators. After participants had rested for 5 min, the staff attached to NHANES measured blood pressure three times using a mercury sphygmomanometer, at 30-s intervals between readings. The final result was the average of three blood pressure readings calculated as systolic blood pressure (SBP) and diastolic blood pressure (DBP). Information was obtained through questionnaires regarding whether participants were taking antihypertensive drugs. According to the definition of the European Society of Cardiology, participants were defined as having hypertension with SBP ≥ 140 mmHg and/or DBP ≥ 90 mmHg^31^, or they were taking antihypertensive drugs. In sensitivity analysis, hypertension was defined as SBP ≥ 130 mmHg and/or DBP ≥ 80 mmHg according to the guideline of hypertension of the American College of Cardiology in 2017^32^ or taking antihypertensive drugs.

### Covariates

Using a Computer-Assisted Personal Interview system, questionnaires, and serum tests, we obtained participant information, including demographics, education level, ratio of family income to poverty, body mass index (BMI), smoking, alcohol use, diabetes, and biochemical data. Demographic data included sex (male, female), age and ethnicity (Hispanic here included Mexican American and other Hispanic, and non-Hispanic here included non-Hispanic white, non-Hispanic black, non-Hispanic Asian, and other ethnicities). Education level is divided into less than high school, high school graduate or general equivalency diploma and more than high school. The value of BMI was calculated as weight in kilograms divided by the square of height in meters. A smoker was defined as a participant who had smoked more than 100 cigarettes during their lifetime. An alcohol user was defined as consuming alcoholic drinks at least once every month in the past year. The definition of a patient with diabetes was being told by a doctor that the participant has diabetes, or taking insulin or oral hypoglycemic agents. The values of alanine aminotransferase (ALT), aspartate aminotransferase (AST), creatinine, urine acid (UA), total cholesterol (TC), and high-density lipoprotein (HDL) cholesterol were obtained by serum testing. Specific detection methods can be found in the laboratory method files of NHANES.

### Statistical analysis

Continuous variables that conformed to the normal distribution are here given as the mean with standard deviation (SD), and those that did not conform to the normal distribution are here given as the median (first quartile, third quartile), while categorical variables are summarized as numbers with percentages. Missing values of BMI, ALT, AST, creatinine, UA, TC, and HDL were replaced by the average of existing data (missing data was less than 1%). PFASs were expressed as continuous variables and quartiles to verify their association with hypertension. The comparison between the hypertension group and non-hypertension group was conducted using the chi-square test for categorical variables, Student’s *t* test for continuous variables that conformed to the normal distribution, and the rank sum test for continuous variables that did not conform to the normal distribution. The association between PFAS exposure and hypertension was evaluated in a multivariable logistic regression model. Before logistic regression, all covariates were screened for collinearity, and no covariate’s variance inflation factor (VIF) was greater than 5. To adjust the confounders stepwise, three models were developed. No variables were adjusted in Model 1. Model 2 was adjusted for demographic data (including sex, age, and ethnicity). In Model 3, in addition to demographic information, the covariates that changed estimates of the effect of PFASs on hypertension by more than 10% or that were significantly associated with hypertension were also adjusted (including sex, age, ethnicity, education level, ratio of family income to the poverty line, BMI, smoking, diabetes, ALT, AST, creatinine, UA, and HDL). To investigate whether the results were modified by sex, age, ethnicity, education level, ratio of family income to the poverty line, BMI, smoking, and diabetes, subgroup analyses and interactive tests were conducted. A spline smoothing plot was used to observe whether there was a linear relationship between PFDeA and blood pressure levels (including SBP and DBP), and the linear regression model was used to verify whether the results were statistically significant.

To test the stability of the results regarding the relationship between PFDeA and hypertension in different situations, several sensitivity analyses were carried out, as follows: (1) restricted to participants who had not taken antihypertensive drugs; (2) using 130/80 mmHg to define hypertension^32^.

The data analyses were carried out using R (http://www.r-project.org; version 3.4.3) and EmpowerStats (http://www.empowerstats.com). The results were considered statistically significant with a *P* value < 0.05.

## Results

### Baseline characteristics

The general characteristics of the study population are shown in Table 1. A total of 4561 participants were included in this study, among whom 1535 had hypertension and 3026 had no hypertension. Compared with participants who did not have hypertension, those with hypertension were older and more likely to be non-Hispanic, to be a smoker, and to have diabetes. Additionally, participants with hypertension had higher levels of BMI, ALT, AST, creatinine, UA, and TC.

**Table 1:** Baseline characteristics of participants (N = 4561).

Number**,** %/mean ± SD/median (Q1, Q3)	Total	Non-Hypertension	Hypertension	*P*
	4561	3026	1535	
Antihypertensive drugs	1127 (24.7)	0 (0.0)	1127 (73.4)	
SBP, mmHg	123.6 ± 17.8	116.2 ± 11.0	138.2 ± 19.7	
DBP, mmHg	70.5 ± 12.3	68.7 ± 10.3	74.0 ± 14.9	
Male	2193 (48.1)	1449 (47.9)	744 (48.5)	0.71
Age, years	47.0 (32.0–61.0)	38.0 (26.0–51.0)	61.0 (52.0–69.0)	< 0.01
Ethnicity				< 0.01
Non-Hispanic	3331 (73.0%)	2179 (72.0)	1152 (75.0)	
Hispanic	1230 (27.0%)	847 (28.0)	383 (25.0)	
Education level				0.02
< High school	1003 (22.0)	620 (20.5)	383 (25.0)	
High school graduate or general equivalency diploma	1064 (23.3)	705 (23.3)	359 (23.4)	
> High school	2491 (54.6)	1698 (56.1)	793 (51.7)	
Unknown	3 (0.1)	3 (0.1)	0 (0.0)	
Ratio of family income to poverty				0.02
≤ 1	911 (20.0)	618 (20.4)	293 (19.1)	
1–3	1700 (37.3)	1120 (37.0)	580 (37.8)	
> 3	1504 (33.0)	1019 (33.7)	485 (31.6)	
Unknown	446 (9.7)	269 (8.9)	177 (11.5)	
BMI, kg/m^2^	29.5 ± 7.2	28.4 ± 6.8	31.5 ± 7.6	< 0.01
Smoking	1862 (40.8)	1087 (35.9)	775 (50.5)	< 0.01
Alcohol use	678 (14.9)	469 (15.5)	209 (13.6)	0.09
Diabetes	651 (14.3)	216 (7.1)	435 (28.3)	< 0.01
ALT, U/L	20.0 (15.0–28.0)	19.0 (15.0–27.0)	21.0 (16.0–29.0)	0.02
AST, U/L	22.0 (18.0–26.0)	21.0 (18.0–26.0)	22.0 (18.0–28.0)	< 0.01
Creatinine, μmol/L	73.4 (61.9–86.6)	71.6 (60.1–84.0)	78.7 (65.4–93.7)	< 0.01
UA, μmol/L	327.2 ± 87.3	315.2 ± 82.0	350.8 ± 92.3	< 0.01
TC, mmol/L	4.9 ± 1.1	4.8 ± 1.0	5.0 ± 1.2	< 0.01
HDL, mmol/L	1.4 ± 0.4	1.4 ± 0.4	1.4 ± 0.5	0.36

#### Association between PFASs and hypertension

The serum levels of six types of PFASs are shown in Table 2. We found higher levels of all PFASs in participants with hypertension in comparison with those who did not have hypertension. Table 3 presents the results of logistic regression analyses for the association between PFASs and hypertension. After fully adjusting for potential confounders, only PFDeA had a significant relationship with hypertension when PFASs were expressed as continuous variables; the OR with 95% CIs for its association with hypertension was 1.2 (1.0–1.4), the *P* value is 0.010. There was no statistically significant correlation between PFAS and hypertension when PFASs were expressed as quartiles (Supplemental Table 1).

**Table 2:** Serum levels of PFASs in different groups (N = 4561).

Number, median (Q1, Q3)	Total	Non-Hypertension	Hypertension	*P*
	4561	3026	1535	
PFDeA, ng/mL	0.2 (0.1–0.3) (mean: 0.3)	0.2 (0.1–0.3) (mean: 0.3)	0.2 (0.1–0.3) (mean: 0.4)	< 0.01
PFHxS, ng/mL	1.2 (0.7–2.1)	1.1 (0.6–1.9)	1.4 (0.8–2.4)	< 0.01
PFNA, ng/mL	0.6 (0.4–0.9)	0.5 (0.3–0.9)	0.7 (0.4–1.1)	< 0.01
n-PFOA, ng/mL	1.5 (1.0–2.4)	1.4 (0.9–2.2)	1.8 (1.1–2.7)	< 0.01
n-PFOS, ng/mL	3.4 (2.0–6.0)	3.0 (1.8–5.1)	4.3 (2.5–8.0)	< 0.01
Sm-PFOS, ng/mL	1.0 (0.8–2.5)	1.2 (0.7–2.1)	2.0 (1.1–3.4)	< 0.01

**Table 3:** Associations of PFASs (ng/mL) with hypertension.

	Model 1	Model 2	Model 3
	OR (95% CI) P	OR (95% CI) P	OR (95% CI) P
PFDeA	1.4 (1.2, 1.6) < 0.01	1.1 (1.0, 1.2) 0.23	1.2 (1.0, 1.4) 0.01
PFHxS	1.1 (1.0, 1.1) < 0.01	1.0 (1.0, 1.0) 0.61	1.0 (0.9, 1.0) 0.54
PFNA	1.4 (1.3, 1.6) < 0.01	1.0 (0.9, 1.1) 0.42	1.0 (0.9, 1.1) 0.64
n-PFOA	1.1 (1.1, 1.1) < 0.01	1.0 (1.0, 1.0) 0.46	1.0 (1.0, 1.0) 0.86
n-PFOS	1.1 (1.1, 1.1) < 0.01	1.0 (1.0, 1.0) 0.01	1.0 (1.0, 1.0) < 0.01
Sm-PFOS	1.4 (1.3, 1.4) < 0.01	1.1 (1.0, 1.1) 0.03	1.1 (1.0, 1.1) 0.05

Model 1: Not adjusted.

Model 2: Adjusted for sex, age, and ethnicity.

Model 3: Adjusted for sex, age, ethnicity, education level, ratio of family income to the poverty line, BMI, smoking, diabetes, ALT, AST, creatinine, UA, and HDL.

#### Subgroup analysis

**Figure 2:**
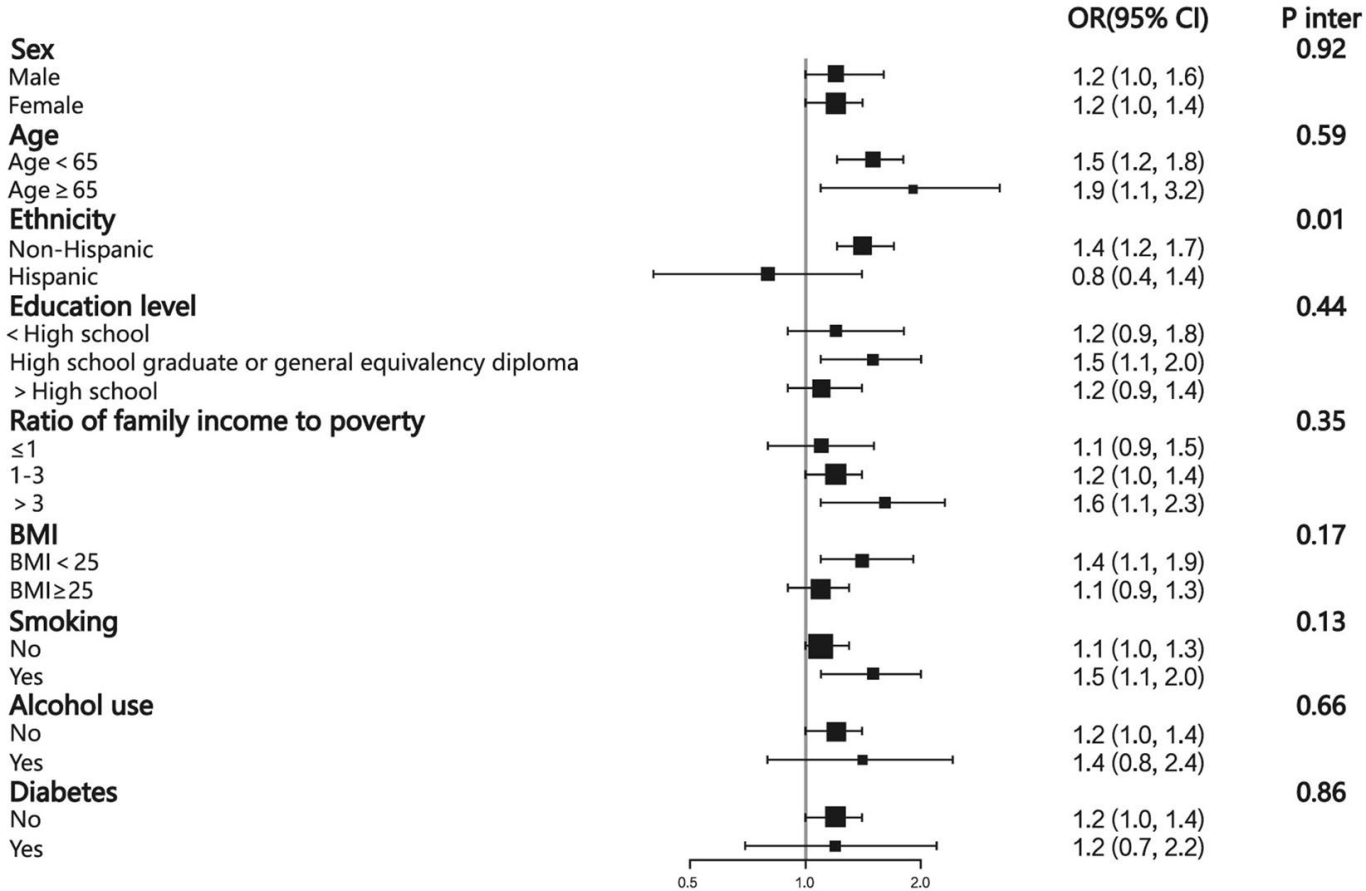
Subgroup analysis of the association between PFDeA (ng/mL) and hypertension. Adjusted for sex, age, ethnicity, education level, ratio of family income to the poverty line, BMI, smoking, diabetes, ALT, AST, creatinine, UA, and HDL, except the subgroup variable. The size of the squares relates to sample size.

In subgroup analyses, it could be observed that the relationship between PFDeA and hypertension was inconsistent in different ethnicities when PFDeA was expressed as a continuous variable. The relationship between PFDeA and hypertension was positive in non-Hispanics but negative in Hispanics, and this result was statistically significant only in non-Hispanics. The *P* value of the interaction test was 0.010 (Fig. 2). A similar result was found when PFDeA was expressed as quartiles, although the *P* value of interaction test did not show statistical significance (Supplemental Table 2).

The association between PFDeA with hypertension according to ethnicity in different regression models is shown in Table 4. After fully adjusting for potential confounders, when PFDeA was expressed as a continuous variable, the association between PFDeA and hypertension for Hispanics was 0.8 (0.4–1.4) and 1.4 (1.2–1.7) for non-Hispanics. When PFDeA was expressed as quartiles, the *P* value for the trend of association between PFDeA and hypertension in Hispanics was 0.5 (0.1–2.0) and 2.5 (1.2–5.1) for non-Hispanics. The subgroup analysis and interactive test showed that the results differed according to ethnicity and there was a stronger correlation in non-Hispanic participants.

**Table 4:** Relationship of PFDeA (ng/mL) and hypertension according to ethnicity.

		Non-Hispanic	Hispanic	P inter
		OR (95% CI) P	OR (95% CI) P	
Model 1	Continuous	1.5 (1.3, 1.8) < 0.01	1.0 (0.8, 1.3) 0.83	0.01
	Quartile			
	Q1	Ref.	Ref.	
	Q2	0.9 (0.7, 1.1) 0.36	0.8 (0.5, 1.1) 0.13	
	Q3	1.0 (0.8, 1.3) 0.76	0.7 (0.5, 1.0) 0.06	
	Q4	1.6 (1.3, 2.0) < 0.01	1.3 (0.9, 1.8) 0.15	
	P trend	6.0 (3.5, 10.1) < 0.01	3.2 (1.2, 8.2) 0.02	0.26
Model 2	Continuous	1.1 (1.0, 1.3) 0.18	0.8 (0.4, 1.3) 0.28	0.14
	Quartile			
	Q1	Ref.	Ref.	
	Q2	0.7 (0.5, 1.0) 0.03	0.8 (0.5, 1.2) 0.26	
	Q3	0.7 (0.6, 0.9) 0.02	0.5 (0.3, 0.7) < 0.01	
	Q4	0.8 (0.7, 1.1) 0.16	0.7 (0.5, 1.1) 0.09	
	P trend	1.0 (0.5, 1.8) 0.94	0.5 (0.2, 1.5) 0.22	0.30
Model 3	Continuous	1.4 (1.2, 1.7) < 0.01	0.8 (0.4, 1.4) 0.39	0.01
	Quartile			
	Q1	Ref.	Ref.	
	Q2	0.8 (0.6, 1.2) 0.31	0.7 (0.4, 1.1) 0.13	
	Q3	1.0 (0.7, 1.3) 0.91	0.5 (0.3, 0.8) < 0.01	
	Q4	1.2 (0.9, 1.6) 0.17	0.7 (0.4, 1.1) 0.11	
	P trend	2.5 (1.2, 5.1) 0.02	0.5 (0.1, 2.0) 0.35	0.05

Model 1: Not adjusted.

Model 2: Adjusted for sex and age.

Model 3: Adjusted for sex, age, education level, ratio of family income to the poverty line, BMI, smoking, diabetes, ALT, AST, creatinine, UA, and HDL.

#### Sensitivity analysis

For participants who identify as non-Hispanic, after restricting to adult participants who did not take antihypertensive drugs and fully adjusting for covariates, the positive association between PFDeA and hypertension was still significant (OR = 1.6, 95% CI: 1.2–2.0). When the definition of hypertension was 130/80 mmHg, the result was also stable (OR = 1.4, 95% CI: 1.1–1.7). For participants who identify as Hispanic, PFDeA did not show a statistically significant correlation with hypertension in any case (Table 5). A similar result was found when PFDeA was expressed as quartiles (Supplemental Table 3).

**Table 5:** Sensitivity analysis of associations between PFDeA (ng/mL) and hypertension.

	Non-Hispanic	Hispanic	P inter
	OR (95% CI) P	OR (95% CI) P	
Restricted to adult participants who did not take antihypertensive drugs (N = 3434)			
	1.6 (1.2, 2.0) < 0.01	1.0 (0.5, 1.9) 0.88	0.04
Using 130/80 mmHg to define hypertension (N = 4561)			
	1.4 (1.1, 1.7) < 0.01	1.0 (0.7, 1.4) 0.95	0.04

Model 1: Not adjusted.

Model 2: Adjusted for sex and age.

Model 3: Adjusted for sex, age, education level, ratio of family income to the poverty line, BMI, smoking, diabetes, ALT, AST, creatinine, UA, and HDL.

#### Association between PFDeA and blood pressure

**Figure 3:**
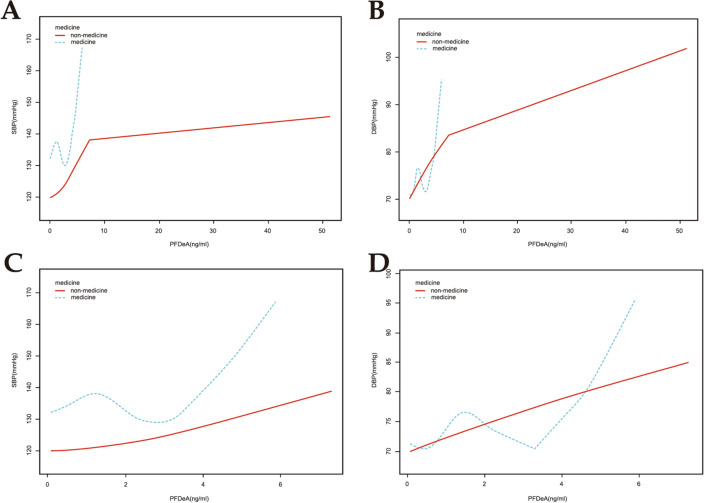
Spline smoothing plot for the relationship between PFDeA (ng/mL) and blood pressure (mmHg) in non-Hispanic participants. (**A**) Trend relationship between PFDeA and systolic blood pressure in all non-Hispanic participants; (**B**) diastolic blood pressure; (**C**, **D**) the same result in participants excluding extreme values. Red line represents non-Hispanic participants who did not take antihypertensive drugs, blue line represents participants who were taking antihypertensive drugs. Adjusted for sex, age, education level, ratio of family income to the poverty line, BMI, smoking, diabetes, ALT, AST, creatinine, UA, and HDL.

Smooth curve fitting was used to test whether there was a linear relationship between PFDeA and blood pressure level (including SBP and DBP) among participants who identify as non-Hispanic. Considering the influence of antihypertensive drugs on blood pressure level, participants were divided into medicine group and non-medicine groups. After the first attempt (Fig. 3A, B), one extreme value of PFDeA was found which seriously affected the result (PFDeA > 50 ng/mL). After removing the extreme value, smooth curve fitting was done again. According to Fig. 3C, D the associations between PFDeA and blood pressure (including SBP and DBP) both had a linear relationship in the non-medicine group. With increased PFDeA, the SBP and DBP levels also increased. However, in the medicine group, there was no obvious linear correlation between PFDeA and blood pressure.

For non-Hispanic participants who did not take antihypertensive drugs (excluding the extreme value above), SBP and DBP roughly accorded with the normal distribution, so linear regression was used to verify the relationship between PFDeA and blood pressure level. The association between PFDeA and blood pressure among non-Hispanic participants who did not take antihypertensive drugs are shown in Table 6. After adjusted all potential confounders in Model 3, the β (95% CI) was SBP 0.7 (0.2–1.1) and DBP 0.8 (0.4–1.2), respectively.

**Table 6:** Associations of PFDeA (ng/mL) with blood pressure (mmHg) in non-Hispanic participants who did not take antihypertensive drugs.

	SBP	DBP
	β (95% CI) P	β (95% CI) P
Model 1	0.9 (0.4, 1.5) < 0.01	0.8 (0.4, 1.2) < 0.01
Model 2	0.5 (0.0, 1.0) 0.04	0.6 (0.2, 1.0) < 0.01
Model 3	0.7 (0.2, 1.1) < 0.01	0.8 (0.4, 1.2) < 0.01

Model 1: Not adjusted.

Model 2: Adjusted for sex and age.

Model 3: Adjusted for sex, age, education level, ratio of family income to the poverty line, BMI, smoking, diabetes, ALT, AST, creatinine, UA, and HDL.

For participants who identify as Hispanic, although the smooth curve fitting seemed to show a linear relationship between PFDeA and blood pressure level (Supplementary Fig. 1), there was no statistically significant correlation between them through linear regression (Supplementary Table 4), regardless of whether the participants take antihypertensive drugs or not.

## Discussion

Previous studies have reported that PFASs greatly affect human health^6–10^. Some studies have pointed out that PFASs are associated with hypertension^20–22,27,28^. However, not all studies have shown the same results^23–26,29^. Most studies were limited to a few types of PFASs, such as PFOA. According to the hypothesis that toxicity increases with the length of the carbon chain^30^, PFDeA may have stronger toxicity than PFOA, but no studies have reported an association between PFDeA and hypertension. In this study, we first found a stable association between PFDeA and hypertension in Americans who identify as non-Hispanic in a cross-sectional study using NHANES data. There was also a positive linear correlation between PFDeA and blood pressure level (both SBP and DBP) in participants who did not take antihypertensive drugs.

Regarding the association between PFASs and hypertension, only a few studies contained PFDeA, and none had a positive result^20,24,25,28^. A cross-sectional study of adolescents from the municipalities of Troms and Balsfjord in Northern Norway reported that some artificial compounds of PFASs were related to hypertension, but PFDeA did not show a significant correlation with hypertension in that study^28^. Another study among participants from China reported similar results^20^. There are also reports that PFDeA as well as other PFASs are not associated with hypertension^24,25^. However, these findings were not consistent with our results, which could be due to the difference in study populations. The participants in these studies were a specific ethnicity in a certain area^20,24,25,28^, which was different from our research where we included many racial and ethnic groups. In addition, previous studies did not consider the influence of liver biochemistry, creatinine, UA, and blood lipids on the results, which are risk factors of cardiovascular disease that would be affected by PFASs^7,8,10,33,34^. In our study, when only demographic factors were adjusted, PFDeA showed no association with hypertension, which was the same as in previous studies. However, after fully adjusting for potential confounders, including sex, age, ethnicity, education level, ratio of family income to the poverty line, BMI, smoking, diabetes, ALT, AST, creatinine, UA, and HDL, PFDeA showed a stable correlation with hypertension.

In this study, we found that ethnicity was an effect modifier between PFDeA and hypertension through subgroup analysis and interactive tests. The baseline characteristics in different ethnicities are shown in Supplemental Table 5. One study showed that the exposure level of PFASs differed in different ethnicities^35^. This was consistent with our finding that the serum level of PFDeA in non-Hispanics was higher than in Hispanics, and the differences were statistically significant (*P* =  < 0.01). Another study reported that non-Hispanics had a higher prevalence of hypertension than Hispanics^36^. This was also consistent with our results: the prevalence of hypertension in non-Hispanics was 34.6%, which was higher than in Hispanics (31.3%) and also higher than the average for all ethnicities (33.7%), and this difference was statistically significant (*P* = 0.03). However, it was worth noting that Hispanics are more likely to have undiagnosed hypertension than other ethnicities^36^. Hispanics have also been found to have a worse hypertension control rate^37^. Therefore, we might underestimate the prevalence of hypertension among Hispanics, which could influence the results. Hispanics and non-Hispanics also had statistically significant differences in education level and ratio of family income to the poverty line (Supplemental Table 5). Access to high-quality clinical care has been found to be very important to hypertensive patients^38^. Hispanics had fewer doctor visitations because of lacking scientific medical knowledge and a lack health insurance among patients^36^. The prevalence of smoking and diabetes and the levels of BMI, ALT, creatinine, UA, HDL all differed between Hispanics and non-Hispanics. All of these might have an impact on the occurrence of hypertension. However, after fully adjusting for confounding factors, including sex, age, education level, ratio of family income to the poverty line, BMI, smoking, diabetes, ALT, AST, creatinine, UA, and HDL, the correlation between PFDeA and hypertension still differed between different ethnicities. The mechanism could not be simply attributed to education level or ratio of family income to the poverty line, even though we initially considered this the most obvious possibility. Owing to the lower level of PFDeA, lower prevalence of hypertension, and poor hypertension control rates, this might mask the influence of PFDeA on hypertension among people who identify as Hispanic. The results of this hidden mechanism might be different. Other factors may also have affected the results, such as genetic and behavioral factors and clinical care. Future studies must be conducted to verify this.

Previous studies have reported that the effect of PFASs on hypertension was inconsistent according to sex^20–22^. A cross-sectional study in China reported a correlation between PFASs and hypertension, and most artificial compounds of PFASs were only related to hypertension in women^20^. Another study among American women also reported a correlation between PFASs and hypertension^21^. However, a cross-sectional study in the Veneto region found that PFASs were only related to hypertension in men^22^. In this study, the association between PFDeA and hypertension was not affected by sex when fully adjusting for covariates. The different results could be explained by the different study participants. Past studies have included particular groups, such as young adults or menopausal women, which differed from our study participants.

Studies have reported that PFASs are related to blood pressure levels^20,22,27,39,40^. However, the results have been inconsistent among studies^23,41^. Only one study on PFASs and blood pressure level included PFDeA, and the results showed that there was no correlation between PFDeA and blood pressure levels^20^. We found a positive correlation between PFDeA and blood pressure levels in non-Hispanic adults who did not take medicine, but no similar results were found in the medicine group. The treatment of hypertension in the medicine group might have mitigated the potential effects of PFDeA on the blood pressure level. Because the type and dosage of antihypertensive drugs were not recorded in the NHANES database, further evaluation could not be conducted. For participants who identify as Hispanic, PFDeA did not show a statistical correlation between blood pressure levels, whether taking antihypertensive drugs or not.

Some studies have found that PFASs exposure will lead to the increase of transaminase and blood lipid levels, the decrease of uric acid levels and the deterioration of renal function.^7–10^, which are also related to hypertension^33,34^. From the clinical experience, these factors are not the intermediary factors of PFAS and hypertension, but as confounding factors, they often affect the results between observation factors and hypertension. Through covariate screening, we also found a statistically significant correlation between these factors and hypertension. However, after adjusting for these covariates in the regression model, PFDeA still showed a stable correlation with hypertension. The biological mechanism underlying the relationship between PFDeA and hypertension might be different. It has been reported that PFASs can affect the signal pathway of peroxisome proliferator activated receptors (PPAR)^42–46^, which is related to cardiovascular disease^47^. Studies have reported that PFASs can activate PPAR and up-regulate the expression of PPAR-α target genes^42–44^. Animal experiments have also reported that PFASs can interfere with the PPAR signaling pathway^45,46^. From this, we infer that PFAS plays an important role in the signal pathway of PPAR, which can explain the effect on hypertension to some extent. Oxidative stress may also be another mechanism by which PFAS affects hypertension^48–53^. It has been reported that PFAS interferes with the production of reactive oxygen species and nitric oxide and weakens the ability of antioxidation in cells by inhibiting catalase and superoxide dismutase^48,49^. Cross-sectional epidemiological studies have also reported that there is a correlation between PFASs exposure and biomarkers of human oxidative stress^50,51^. Although oxidative stress is not the only cause of hypertension, it does play an important role^52,53^. This shows that PFAS can affect the balance of reactive oxygen species and nitric oxide, which leads to oxidative stress, which in turn may be another important mechanism by which PFASs affect blood pressure. Another explanation is that PFASs will affect the signal pathway of thyroid hormone production^54–57^. This also has a large impact on the occurrence of hypertension^58^. Studies have shown that PFAS can affect the expression of thyroid hormone-related genes, destroy the structure of thyroid follicular cells, and increase the metabolic clearance rate of thyroid-related hormones, thus reducing the circulating level of thyroid hormones^54,55^. Epidemiological studies have also shown that PFAS exposure will increase the risk of subclinical hypothyroidism^56,57^. There is also a statistically significant correlation between subclinical hypothyroidism and elevated blood pressure^58^. In this way, PFAS exposure leads to the destruction of thyroid structure and the increases the metabolic clearance rate of thyroid hormones, which reduces the level of circulating thyroid hormones and so increases the risk of subclinical hypothyroidism, which may be another explanation for the influence of PFAS on blood pressure level.

As a widely used artificial PFAS compound, PFOA has been reported to have a large influence on human health^2,3,6^, it has therefore been listed for restricted management^3^. In recent years, PFOA has been gradually replaced by other types of PFASs, including some PFASs with long carbon chains^59^, but long-chain PFASs have longer half-lives and stronger toxicity than those with short chains^30,60^. Compared with perfluorobutanoic acid, PFHxA and other short-chain PFASs, PFOA has a longer half-life of 8.5 years. One artificial compound of long-chain PFAS, PFDeA, is gradually deposited in the environment over time^3^; to our knowledge, the present study is the first to report an association between PFDeA and hypertension, which is bound to affect human health. Our findings highlight the importance of monitoring not only PFOA but also other PFASs that may affect human health, such as PFDeA.

There were some limitations to our study. The research population in this study came from NHANES, which was included by multi-stage stratified sampling. But only one-third of NHANES participants from 2013 to 2018 had PFAS measurement data. Therefore, this inevitably causes a certain selection bias. And NHANES only included individuals, so, the results are not generalizable to other countries. Because human blood pressure would fluctuate at different times, some healthy participants were misjudged as patients with hypertension. Some participants took antihypertensive drugs due to other diseases, such as hyperthyroidism patients taking beta blockers, and these participants were also misjudged as hypertensive patients. These increased the risk of making an error of the first kind in statistically. Furthermore, we could not determine a causal relationship in this cross-sectional study. At present, the biological pathway of PFDeA is not clear, and the further study of the biological pathway in the future may be helpful to explain the reasons for the influence on blood pressure. In addition, the mechanism of PFDeA affecting blood pressure remains unclear; further studies are needed to clarify the present results.

## Conclusions

The present study showed that increased PFDeA was associated with a high prevalence of hypertension in non-Hispanic Americans. Moreover, there was a positive association between serum PFDeA concentration and blood pressure level in non-Hispanic American adults who did not take antihypertensive drugs.

### Supplementary Information

Supplementary Information.

## Data Availability

The study data were derived from NHANES which can be obtained online (https://www.cdc.gov/nchs/nhanes/index.htm). The study protocol of NHANES has been approved by the Ethics Review Board of the National Center for Health Statistics Research. All participants provided written informed consent to authorize the use of their data.
