# Supplementary material for: Association of PFDeA exposure with hypertension (NHANES, 2013–2018)
Source: Sci Rep. 2024 Jan 9;14:918. doi: 10.1038/s41598-024-51187-4 (PMC10776849; doi:10.1038/s41598-024-51187-4)
Supplement: Supplementary file 1 — Supplementary Information. [file 41598_2024_51187_MOESM1_ESM.docx]

**Supplemental Table 1. Associations of PFASs (ng/mL) with hypertension(Quartile)**

|  | **Model 1** | **Model 2** | **Model 3** |
| --- | --- | --- | --- |
|  | OR (95% CI) P | OR (95% CI) P | OR (95% CI) P |
| **PFDeA** |  |  |  |
| Q1 | Ref. | Ref. | Ref. |
| Q2 | 0.8 (0.7, 1.0) 0.11 | 0.7 (0.6, 0.9) 0.01 | 0.8 (0.6, 1.0) 0.07 |
| Q3 | 0.9 (0.8, 1.1) 0.45 | 0.6 (0.5, 0.8) <0.01 | 0.8 (0.6, 1.0) 0.10 |
| Q4 | 1.5 (1.3, 1.8) <0.01 | 0.8 (0.6, 1.0) 0.03 | 1.0 (0.8, 1.3) 0.81 |
| P trend | 5.3 (3.4, 8.4) <0.01 | 0.8 (0.5, 1.4) 0.50 | 1.7 (0.9, 3.1) 0.11 |
| **PFHxS** |  |  |  |
| Q1 | Ref. | Ref. | Ref. |
| Q2 | 1.5 (1.3, 1.9) <0.01 | 1.0 (0.8, 1.3) 0.75 | 1.1 (0.9, 1.5) 0.31 |
| Q3 | 1.9 (1.6, 2.3) <0.01 | 0.9 (0.7, 1.2) 0.52 | 1.0 (0.8, 1.3) 0.94 |
| Q4 | 2.3 (1.9, 2.7) <0.01 | 0.9 (0.7, 1.1) 0.32 | 1.0 (0.7, 1.3) 0.86 |
| P trend | 1.3 (1.2, 1.4) <0.01 | 0.9 (0.9, 1.0) 0.20 | 1.0 (0.9, 1.1) 0.49 |
| **PFNA** |  |  |  |
| Q1 | Ref. | Ref. | Ref. |
| Q2 | 1.3 (1.1, 1.6) <0.01 | 1.0 (0.8, 1.3) 0.71 | 1.1 (0.8, 1.4) 0.54 |
| Q3 | 1.8 (1.5, 2.1) <0.01 | 1.1 (0.9, 1.4) 0.26 | 1.2 (1.0, 1.6) 0.12 |
| Q4 | 2.4 (2.0, 2.8) <0.01 | 1.0 (0.8, 1.2) 0.96 | 1.1 (0.9, 1.4) 0.34 |
| P trend | 2.3 (2.0, 2.7) <0.01 | 1.0 (0.8, 1.2) 0.89 | 1.1 (0.9, 1.4) 0.41 |
| **n-PFOA** |  |  |  |
| Q1 | Ref. | Ref. | Ref. |
| Q2 | 1.2 (1.0, 1.4) 0.06 | 1.0 (0.8, 1.2) 0.71 | 1.0 (0.8, 1.3) 0.79 |
| Q3 | 1.4 (1.2, 1.7) <0.01 | 0.8 (0.7, 1.0) 0.08 | 0.9 (0.7, 1.2) 0.47 |
| Q4 | 2.1 (1.8, 2.5) <0.01 | 0.9 (0.8, 1.2) 0.60 | 1.1 (0.8, 1.4) 0.49 |
| P trend | 1.3 (1.3, 1.4) <0.01 | 1.0 (0.9, 1.1) 0.71 | 1.0 (0.9, 1.1) 0.43 |
| **n-PFOS** |  |  |  |
| Q1 | Ref. | Ref. | Ref. |
| Q2 | 1.2 (1.0, 1.4) 0.12 | 0.9 (0.7, 1.1) 0.35 | 1.0 (0.8, 1.3) 0.91 |
| Q3 | 1.6 (1.4, 2.0) <0.01 | 0.9 (0.7, 1.1) 0.38 | 1.1 (0.8, 1.4) 0.60 |
| Q4 | 2.9 (2.4, 3.5) <0.01 | 1.1 (0.9, 1.3) 0.53 | 1.3 (1.0, 1.7) 0.05 |
| P trend | 1.1 (1.1, 1.2) <0.01 | 1.0 (1.0, 1.0) 0.18 | 1.0 (1.0, 1.1) 0.02 |
| **Sm-PFOS** |  |  |  |
| Q1 | Ref. | Ref. | Ref. |
| Q2 | 1.1 (0.9, 1.4) 0.20 | 0.9 (0.7, 1.1) 0.40 | 0.9 (0.7, 1.1) 0.25 |
| Q3 | 1.7 (1.4, 2.0) <0.01 | 0.8 (0.6, 1.0) 0.07 | 0.8 (0.6, 1.0) 0.11 |
| Q4 | 3.6 (3.0, 4.4) <0.01 | 1.0 (0.8, 1.3) 0.89 | 1.0 (0.8, 1.3) 0.88 |
| P trend | 1.5 (1.5, 1.6) <0.01 | 1.0 (1.0, 1.1) 0.38 | 1.0 (1.0, 1.1) 0.28 |

Model 1: Not adjusted

Model 2: Adjusted for sex, age, and ethnicity

Model 3: Adjusted for sex, age, ethnicity, education level, ratio of family income to the poverty line, BMI, smoking, diabetes, ALT, AST, creatinine, UA, and HDL

**Supplemental Table 2.Subgroup analysis of the association between PFDeA (ng/mL) and hypertension(Quartile)**

|  | OR (95% CI) P | | | | P trend | P inter |
| --- | --- | --- | --- | --- | --- | --- |
|  | Q1 | Q2 | Q3 | Q4 |  |  |
| **Sex** | | | | | | 0.29 |
| Male | Ref. | 0.7 (0.5, 1.1) | 0.9 (0.6, 1.2) | 0.9 (0.6, 1.3) | 0.76 |  |
| Female | Ref. | 0.8 (0.5, 1.2) | 0.7 (0.5, 1.0) | 1.1 (0.8, 1.6) | 0.07 |  |
| **Age** | | | | | | 0.28 |
| Age＜65 | Ref. | 0.9 (0.7, 1.2) | 1.0 (0.8, 1.3) | 1.7 (1.3, 2.2) | <0.01 |  |
| Age ≥ 65 | Ref. | 0.7 (0.4, 1.3) | 1.0 (0.6, 1.8) | 1.2 (0.7, 2.2) | 0.06 |  |
| **Ethnicity** | | | | | | 0.05 |
| Non-Hispanic | Ref. | 0.8 (0.6, 1.2) | 1.0 (0.7, 1.3) | 1.2 (0.9, 1.6) | 0.02 |  |
| Hispanic | Ref. | 0.7 (0.4, 1.1) | 0.5 (0.3, 0.8) | 0.7 (0.4, 1.1) | 0.35 |  |
| **Education level** | | | | | | 0.98 |
| <High school | Ref. | 0.8 (0.6, 1.2) | 1.0 (0.7, 1.3) | 1.2 (0.9, 1.6) | 0.65 |  |
| High school graduate or general equivalency diploma | Ref. | 0.7 (0.4, 1.1) | 0.5 (0.3, 0.8) | 0.7 (0.4, 1.1) | 0.36 |  |
| ＞High school | Ref. | 0.8 (0.6, 1.2) | 1.0 (0.7, 1.3) | 1.2 (0.9, 1.6) | 0.21 |  |
| **Ratio of family income to poverty** | | | | | | 0.96 |
| ≤1 | Ref. | 0.9 (0.5, 1.5) | 0.8 (0.5, 1.3) | 1.1 (0.6, 1.8) | 0.63 |  |
| 1–3 | Ref. | 0.7 (0.5, 1.0) | 0.8 (0.5, 1.1) | 1.0 (0.7, 1.5） | 0.21 |  |
| ＞3 | Ref. | 0.9 (0.5, 1.5) | 0.9 (0.6, 1.5) | 1.1 (0.7, 1.7) | 0.27 |  |
| **BMI** | | | | | | 0.64 |
| BMI＜25 | Ref. | 0.7 (0.3, 1.4) | 0.9 (0.5, 1.7) | 1.0 (0.5, 1.8) | 0.47 |  |
| BMI≥25 | Ref. | 0.8 (0.6, 1.0) | 0.7 (0.6, 1.0) | 1.0 (0.7, 1.2) | 0.42 |  |
| **Smoking** | | | | | | 0.21 |
| No | Ref. | 0.8 (0.5, 1.2) | 0.9 (0.6, 1.3) | 1.2 (0.8, 1.7) | 0.04 |  |
| Yes | Ref. | 0.7 (0.5, 1.1) | 0.8 (0.5, 1.1) | 0.9 (0.6, 1.3) | 0.87 |  |
| **Alcohol use** | | | | | | 0.26 |
| No | Ref. | 0.7 (0.6, 1.0) | 0.7 (0.6, 1.0) | 1.0 (0.8, 1.3) | 0.07 |  |
| Yes | Ref. | 1.1 (0.4, 2.8) | 1.3 (0.6, 3.0) | 1.0 (0.4, 2.4) | 0.67 |  |
| **Diabetes** | | | | | | 0.92 |
| No | Ref. | 0.8 (0.6, 1.1) | 0.8 (0.6, 1.1) | 1.1 (0.8, 1.4) | 0.13 |  |
| Yes | Ref. | 0.7 (0.4, 1.3) | 0.8 (0.4, 1.5) | 1.0 (0.5, 1.7) | 0.54 |  |

Adjusted for sex, age, ethnicity, education level, ratio of family income to the poverty line, BMI, smoking, diabetes, ALT, AST, creatinine, UA, and HDL, except the subgroup variable.

**Supplemental Table 3.Sensitivity analysis of associations between PFDeA (ng/mL) and hypertension(Quartile)**

|  | **Non-Hispanic** | **Hispanic** | **P** inter |
| --- | --- | --- | --- |
|  | OR (95% CI) P | OR (95% CI) P |  |
| **Restricted to adult participants who did not take antihypertensive drugs (N = 3434)** | | | |
| Q1 | Ref. | Ref. |  |
| Q2 | 0.8 (0.5, 1.3) 0.37 | 0.9 (0.4, 1.7) 0.71 |  |
| Q3 | 1.1 (0.7, 1.7) 0.71 | 0.6 (0.3, 1.2) 0.14 |  |
| Q4 | 1.3 (0.9, 2.0) 0.19 | 0.7 (0.3, 1.4) 0.28 |  |
| P trend | 3.2 (1.1, 9.4) 0.03 | 0.4 (0.1, 2.5) 0.32 | 0.06 |
| **Using 130/80 mmHg to define hypertension (N = 4561)** | | | |
| Q1 | Ref. | Ref. |  |
| Q2 | 0.9 (0.6, 1.1) 0.30 | 1.4 (0.9, 2.1) 0.16 |  |
| Q3 | 1.0 (0.7, 1.3) 0.81 | 0.9 (0.6, 1.3) 0.54 |  |
| Q4 | 1.2 (0.9, 1.6) 0.16 | 1.2 (0.8, 1.9) 0.37 |  |
| P trend | 2.3 (1.2, 4.4) 0.02 | 1.3 (0.4, 4.2) 0.69 | 0.41 |

Model 1: Not adjusted

Model 2: Adjusted for sex and age

Model 3: Adjusted for sex, age, education level, ratio of family income to the poverty line, BMI, smoking, diabetes, ALT, AST, creatinine, UA, and HDL

**Supplemental Figure 1. Spline smoothing plot for the relationship between PFDeA (ng/mL) and blood pressure (mmHg) in Hispanic participants**


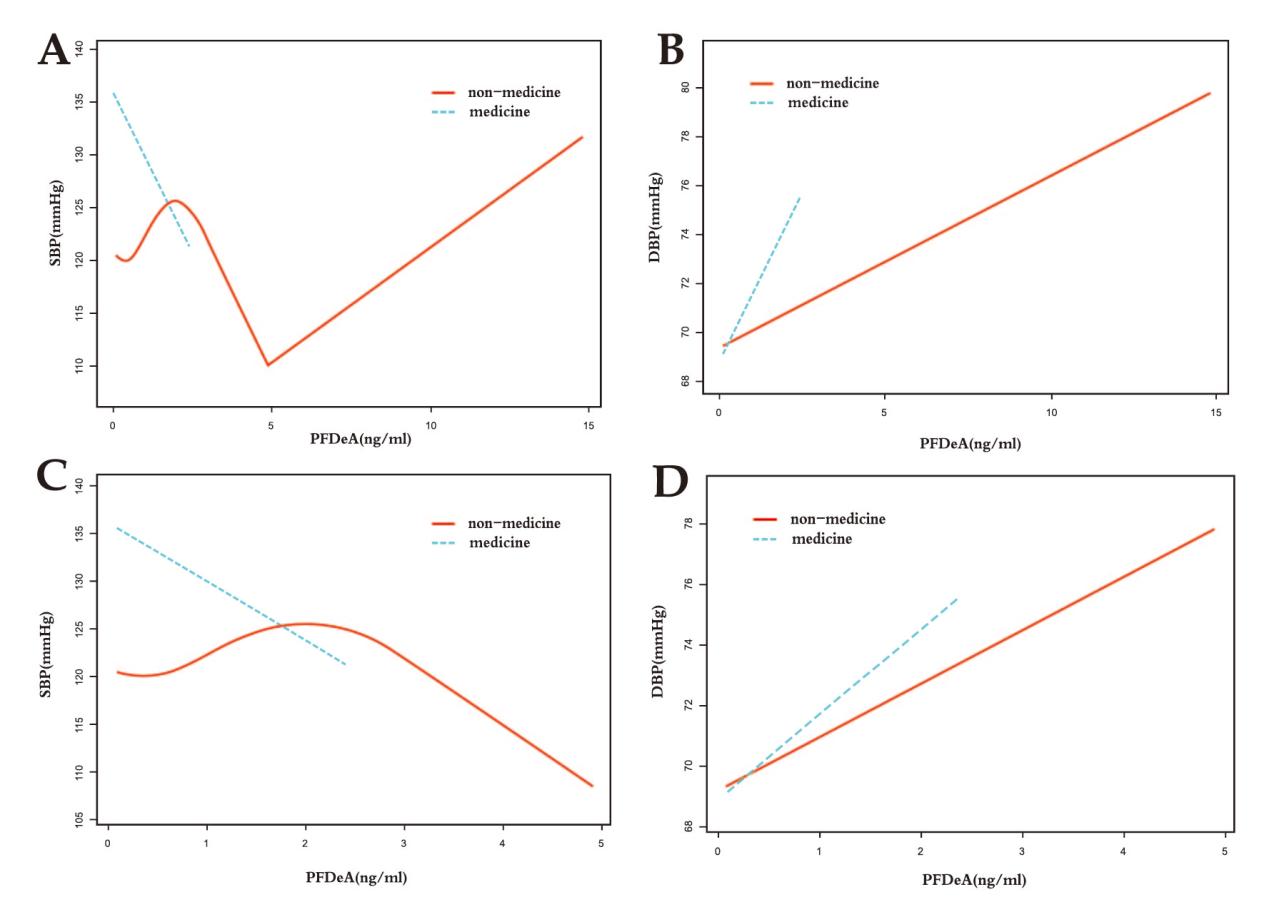


1. Trend relationship between PFDeA and systolic blood pressure in all Hispanic participants; B) diastolic blood pressure; C, D) the same result in participants excluding extreme values. Red line represents Hispanic participants who did not take antihypertensive drugs, blue line represents participants who were taking antihypertensive drugs.

Adjusted for sex, age, education level, ratio of family income to the poverty line, BMI, smoking, diabetes, ALT, AST, creatinine, UA, and HDL.

**Supplemental Table 4. Associations of PFDeA (ng/mL) with blood pressure (mmHg) in Hispanic participants (N =1229)**

|  | **Non-Medicine** | **Medicine** |
| --- | --- | --- |
|  | β (95% CI) P | β (95% CI) P |
| **SBP** | 0.7 (-2.7, 4.2) 0.681 | -8.1 (-20.1, 4.0) 0.192 |
| **DBP** | 1.4 (-1.4, 4.3) 0.324 | 4.9 (-2.2, 11.9) 0.178 |

Adjusted for sex, age, education level, ratio of family income to the poverty line, BMI, smoking, diabetes, ALT, AST, creatinine, UA, and HDL

**Supplemental Table 5. Baseline characteristics of participants in different** ethnicities**(N =4561)**

|  | Total | Non-Hispanic | Hispanic | P |
| --- | --- | --- | --- | --- |
| Number, % / mean ± SD / median (Q1, Q3) | 4561 | 3331 | 1230 |  |
| **Hypertension** | 1535 (33.7) | 1152 (34.6) | 383 (31.1) | 0.03 |
| **Antihypertensive drugs** | 1127 (24.7) | 862 (25.9) | 265 (21.5) | 0.03 |
| **SBP, mmHg** | 123.6 ± 17.8 | 123.7 ± 17.8 | 123.5 ± 17.9 | 0.75 |
| **DBP, mmHg** | 70.5 ± 12.3 | 70.8 ± 12.4 | 69.5 ± 11.9 | <0.01 |
| **Male** | 2193 (48.1) | 1626 (48.8) | 567 (46.1) | 0.10 |
| **Age, years** | 47.0 (32.0-61.0) | 47.0 (32.0-61.0) | 46.0 (31.2-61.0) | 0.20 |
| **Education level** | | | | <0.01 |
| <High school | 1003 (22.0) | 491 (14.7) | 512 (41.6) |  |
| High school graduate or general equivalency diploma | 1064 (23.3) | 804 (24.1) | 260 (21.1) |  |
| ＞High school | 2491 (54.6) | 2034 (61.1) | 457 (37.2) |  |
| Unknow | 3 (0.1) | 2 (0.1) | 1 (0.1) |  |
| **Ratio of family income to poverty** | | | | <0.01 |
| ≤1 | 911 (20.0) | 601 (18.0) | 310 (25.2) |  |
| 1–3 | 1700 (37.3) | 1198 (36.0) | 502 (40.8) |  |
| ＞3 | 1504 (33.0) | 1253 (37.6) | 251 (20.4) |  |
| Unkonw | 446 (9.8) | 279 (8.4) | 167 (13.6) |  |
| **BMI, kg/m^2^** | 29.5 ± 7.2 | 29.2 ± 7.5 | 30.2 ± 6.4 | <0.01 |
| **Smoking** | 1862 (40.8) | 1446 (43.4) | 416 (33.8) | <0.01 |
| **Alcohol use** | 678 (14.9) | 517 (15.5) | 161 (13.1) | 0.04 |
| **Diabetes** | 651 (14.3) | 439 (13.2) | 212 (17.2) | <0.01 |
| **ALT, U/L** | 20.0 (15.0-28.0) | 19.0 (15.0-27.0) | 22.0 (16.0-31.0) | <0.01 |
| **AST, U/L** | 22.0 (18.0-26.0) | 21.0 (18.0-26.0) | 22.0 (18.0-27.0) | <0.01 |
| **Creatinine, μmol/L** | 73.4 (61.9-86.6) | 76.0 (64.5-89.3) | 66.3 (56.6-79.6) | <0.001 |
| **UA, μmol/L** | 327.2 ± 87.3 | 330.9 ± 86.8 | 317.1 ± 87.9 | <0.01 |
| **TC, mmol/L** | 4.9 ± 1.1 | 4.9 ± 1.1 | 4.9 ± 1.1 | 0.13 |
| **HDL, mmol/L** | 1.4 ± 0.4 | 1.4 ± 0.4 | 1.3 ± 0.4 | <0.01 |
| **PFDeA, ng/mL** | 0.2 (0.1-0.3)  (mean: 0.3) | 0.2 (0.1-0.3)  (mean: 0.3) | 0.2 (0.1-0.2)  (mean: 0.2) | <0.01 |
| **PFHxS, ng/mL** | 1.2 (0.7-2.1) | 1.3 (0.7-2.2) | 1.1 (0.6-1.8) | <0.01 |
| **PFNA, ng/mL** | 0.6 (0.4-0.9) | 0.6 (0.4-1.0) | 0.5 (0.3-0.8) | <0.01 |
| **n-PFOA, ng/mL** | 1.5 (1.0-2.4) | 1.6 (1.0-2.5) | 1.4 (0.9-2.0) | <0.01 |
| **n-PFOS, ng/mL** | 3.4 (2.0-6.0) | 3.7 (2.1-6.7) | 2.8 (1.7-4.6) | <0.01 |
| **Sm-PFOS, ng/mL** | 1.4 (0.8-2.5) | 1.5 (0.8-2.7) | 1.2 (0.7-2.2) | <0.01 |
